# Triage Performance of *FAM19A4/miR124‐2* Methylation: 18‐Month Follow‐Up Results From a Prospective Observational Study Within the Dutch Primary HPV‐Based Cervical Screening Program

**DOI:** 10.1002/ijc.70626

**Published:** 2026-06-29

**Authors:** Lisanne Verhoef, Mila S. Griffioen, Maaike C. G. Bleeker, John W. J. Hinrichs, Albert G. Siebers, Albertus T. Hesselink, Chris J. L. M. Meijer, Renske D. M. Steenbergen, Johannes Berkhof, Daniëlle A. M. Heideman

**Affiliations:** ^1^ Pathology, Amsterdam UMC Vrije Universiteit Amsterdam Amsterdam the Netherlands; ^2^ Imaging and Biomarkers Cancer Center Amsterdam Amsterdam the Netherlands; ^3^ Symbiant Pathology Hoorn the Netherlands; ^4^ Palga Foundation: The Dutch Nationwide Pathology Databank Houten the Netherlands; ^5^ Self‐Screen B.V. Amsterdam the Netherlands; ^6^ Epidemiology and Data Science, Amsterdam UMC Vrije Universiteit Amsterdam Amsterdam the Netherlands; ^7^ Department of Pathology and Medical Biology, University Medical Center Groningen University of Groningen Groningen the Netherlands

**Keywords:** cervical cancer, cervical intraepithelial neoplasia, cervical screening: human papillomavirus, DNA methylation

## Abstract

DNA methylation analysis of the genes *FAM19A4* and *miR124‐2* has emerged as a promising triage strategy for high‐risk (hr) human papillomavirus (HPV)‐positive women in cervical screening. This study reports a prospective evaluation of the diagnostic accuracy of *FAM19A4/miR124‐2* methylation in a large population‐based primary HPV‐based screening cohort with 18 months of follow‐up. In this prospective observational study, clinician‐collected cervical samples from 3848 consecutive hrHPV‐positive women who participated in the Dutch national primary HPV‐based screening program were tested with the QIAsure Methylation Test, a quantitative methylation‐specific PCR assessing *FAM19A4* and *miR124‐2* methylation. Through linkage to the Dutch Nationwide Pathology Databank (Palga), 11 cases of cervical carcinoma, 293 cervical intraepithelial neoplasia grade 3 (CIN3), and 29 adenocarcinoma in situ (AIS) were identified during 18 months of follow‐up. Clinical performance for CIN3, AIS, and cancer (CIN3+) detection was assessed based on (I) the threshold of the QIAsure Methylation Test, and (II) thresholds corresponding with clinical specificities of 70% and 80% in this hrHPV‐positive study population. The sensitivity and specificity of *FAM19A4/miR124‐2* methylation for CIN3+ detection using the QIAsure Methylation Test threshold (I) were 57.4% (95% CI: 52.0–62.7) and 88.3% (95% CI: 87.2–89.5), respectively. At predefined specificities of 70% and 80%, *FAM19A4/miR124‐2* methylation (II) demonstrated a CIN3+ sensitivity of 75.4% (95% CI: 70.7–80.0) and 66.1% (95% CI: 61.0–71.2), respectively. In conclusion, *FAM19A4/miR124‐2* methylation analysis showed good triage performance in a primary HPV‐based screening setting. These findings support the use of methylation‐based testing as a triage strategy in primary HPV‐based screening programs, with an appropriate follow‐up policy for hrHPV‐positive women who test methylation‐negative.

## What's New?

*FAM19A4/miR124‐2* DNA methylation analysis has extensively been evaluated in clinical studies, but not yet on primary HPV‐based screening cohorts. Here, the authors evaluated the diagnostic accuracy of *FAM19A4/miR124‐2* methylation on 3,848 consecutive hrHPV‐positive clinician‐collected cervical samples from women who participated in the Dutch primary HPV‐based cervical screening program. Their findings support the use of methylation‐based triage testing in primary HPV‐based screening programs, provided there is an adequate follow‐up policy in place for hrHPV‐positive, methylation‐negative women.

## Graphical abstract

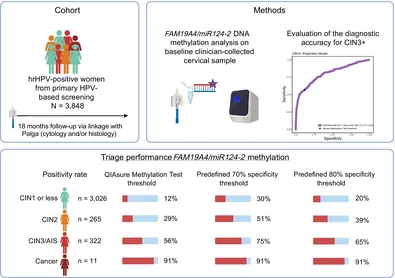

**Abbreviations**
ACTB: β‐actin
ADC: adenocarcinoma
AIS: adenocarcinoma in situ
ASC‐US: atypical squamous cells of undetermined significance
AUC: area under the curve
CI: confidence intervals
CIN: cervical intraepithelial neoplasia
CIN3+: CIN3 and cancer
Ct: cycle threshold
HPV: human papillomavirus
hrHPV: high‐risk human papillomavirus
HSIL: high‐grade squamous intraepithelial lesion
LSIL: low‐grade squamous intraepithelial lesion
NILM: negative for intraepithelial lesion or malignancy
NPV: negative predictive value
Palga: Dutch Nationwide Pathology Databank
PPV: positive predictive value
ROC: receiver operating characteristic
SSC: squamous cell carcinoma
WHO: World Health Organization

## 1 Introduction

Cervical cancer is the most common gynecological malignancy worldwide and is primarily caused by a persistent infection with high‐risk (hr) human papillomavirus (HPV). Cervical cancer is preventable through HPV prophylactic vaccination and cervical screening. The World Health Organization (WHO) and European guidelines recommend HPV testing as the primary screening method, given its superior sensitivity compared to cytology [1, 2, 3, 4, 5, 6]. In settings where primary HPV screening has been implemented, cytology is often used as a triage test for hrHPV‐positive women to mitigate the risks of over‐referral and potential overtreatment. Results from the Dutch cervical screening program indicate that, following the transition from cytology‐based to HPV‐based screening with cytology triage in 2017, approximately 30% more cervical intraepithelial neoplasia (CIN) grade 2 and worse (CIN2+) were detected, but at the cost of more than twice as many CIN1 or less [7, 8]. Furthermore, considerable variability in the diagnostic performance of cytology has been reported [1, 9]. Its effectiveness depends on a well‐organized infrastructure, including access to trained cytopathologists and cytotechnicians, as well as resources that may be under pressure or even scarce in remote or underserved regions.

While HPV genotyping can be used alongside cytology as an additional triage step, DNA methylation markers are emerging as a potential alternative to cytology offering an objective triage approach for hrHPV‐positive women. To date, several host‐cell DNA methylation panels have been developed of which the *FAM19A4/miR124‐2* methylation test (QIAsure Methylation Test, QIAGEN, Hilden, Germany) has been most extensively evaluated in clinical studies [10, 11]. In an international study, the *FAM19A4/miR124‐2* methylation test has demonstrated a high intra‐ and inter‐laboratory agreement [12]. High methylation levels have been associated with cervical cancer and advanced CIN lesions (i.e., defined by a 5‐year or longer hrHPV infection) and a worldwide study confirmed that the *FAM19A4/miR124‐2* methylation test detects nearly all cervical cancers (98.3%; 510/519) [13, 14]. In an European multicenter retrospective study, the methylation markers *FAM19A4* and *miR124‐2* demonstrated a sensitivity of 78.6% and a specificity of 76.8% for detecting CIN3+ in hrHPV‐positive clinician‐collected cervical screening samples [15], consistent with other studies (sensitivities 67%–78%, specificities 68%–94%) [16, 17, 18, 19]. Additionally, *FAM19A4/miR124‐2* methylation‐negativity is associated with clinical regression of CIN2/3 lesions [20]. A 14‐year follow‐up study shows that *FAM19A4/miR124‐2* methylation‐negative hrHPV‐positive women have a lower risk of cervical cancer and a CIN3+ risk comparable to hrHPV‐positive women with normal cytology [18, 21, 22].

Methylation‐based triage by *FAM19A4/miR124‐2* methylation is therefore worth considering in routine primary HPV‐based cervical screening. This report presents the first prospective evaluation of the diagnostic accuracy of *FAM19A4/miR124‐2* methylation in a large population‐based primary HPV‐based screening cohort with 18 months of follow‐up.

## 2 Methods

### 2.1 Study Population

This prospective observational study encompassed clinician‐collected cervical samples derived from hrHPV‐positive women aged between 30 and 60 years, who participated in the Dutch HPV‐based cervical screening program in the period December 2020 to April 2021. The screening samples were consecutively collected at Symbiant Pathology (Hoorn, the Netherlands), which is one of the designated screening facilities within the Netherlands. An aliquot was extracted from each sample between 1 and 8 weeks after collection (median: 1.5 weeks), following the completion of the primary HPV test and cytology triage, and subsequently stored at 4°C until further analysis (median storage of 6 weeks, range: 1–13 weeks).

### 2.2 Screening and Follow‐Up Procedures

The Netherlands implemented primary HPV‐based cervical screening with cytology triage in 2017. For participants in this study, this was their first screening round using a primary HPV test. Clinician‐collected cervical samples were collected in ThinPrep PreservCyt Solution (Hologic, Marlborough, MA, US) for hrHPV DNA testing and, when applicable, subsequent reflex liquid‐based cytology.

Presence of hrHPV DNA was assessed with the fully automated Cobas HPV Test (Cobas 4800 System, Roche Molecular systems, Branchburg, NJ, US) [23]. The Cobas HPV Test features automated sample preparation combined with real‐time PCR technology to detect 14 hrHPV genotypes (i.e., HPV 16/18/31/33/35/39/45/51/52/56/58/59/66/68). The assay analyses three separate channels for hrHPV DNA detection, that is, HPV16, HPV18, and a pool of 12 remaining hrHPV types. A sample is considered hrHPV‐positive if at least one HPV channel yields a cycle threshold (Ct) value below its predefined cutoff. At the time of this study, HPV genotype information was not used in the management of hrHPV‐positive women within the Dutch screening program, and only the overall HPV result was recorded.

In accordance with routine screening procedures implemented in 2017 and in place at the time of this study, cytology is classified according to the CISOE‐A system [24] which is automatically translated into the Bethesda score. Women positive for hrHPV were referred for colposcopy if baseline cytology showed atypical squamous cells of undetermined significance (ASC‐US) or worse. Women with normal baseline cytology (i.e., negative for intraepithelial lesion or malignancy, NILM) were re‐invited for repeat cytology after 6 months and referred for colposcopy if ASC‐US or worse.

Histological outcomes were based on the most severe lesion detected and categorized as no CIN, CIN1, CIN2, CIN3, adenocarcinoma in situ (AIS) or invasive cervical cancer, including adenocarcinoma (ADC) and squamous cell carcinoma (SCC) [25]. Women with a non‐diagnostic histology result were categorized as “no CIN.” Women without a histological outcome, including women with only cytological follow‐up after 18 months, were classified as “no histology.” The cytological and histological outcomes were obtained from the Dutch Nationwide Pathology Databank (Palga), with follow‐up information collected until October 2022 (median of 20 months after study enrolment, range 18–23 months).

### 2.3 *FAM19A4/miR124‐2* Methylation Analysis

Bisulfite conversion was performed using a direct conversion protocol on the clinician‐collected cervical samples (Epitect Fast 96 Bisulfite conversion kit; QIAGEN, Hilden, Germany) [26]. Bisulfite‐converted DNA was subsequently used as a template for DNA methylation analysis targeting *FAM19A4* and *miR124‐2* genes using the CE‐marked in vitro diagnostic (IVD) QIAsure Methylation Test (QIAGEN, Hilden, Germany). Methylation analysis was conducted on the Rotorgene Q MDX 5plex HRM instrument (QIAGEN, Hilden, Germany) according to the manufacturer's instructions. A sample was considered valid for methylation analysis when β‐actin (*ACTB*) had a Ct value below 26.4.

### 2.4 Data and Statistical Analysis

Samples were excluded if they did not represent a primary screening sample, had inadequate cytology results for triage cytology, or lacked sufficient residual material for valid methylation analysis. Additionally, one sample was excluded due to the presence of a non‐cervical gynecological tumor at the time of cervical screening.

Methylation levels were expressed as ΔΔCt ratios that were obtained using the comparative Ct method (2^−ΔΔ*Ct*^ × 100) [27]. Methylation levels were visualized and compared between histological subgroups by constructing boxplots of the log_2_‐transformed ΔΔCt ratios. Differences between categories were assessed with the Kruskal–Wallis test followed by pairwise post hoc Mann–Whitney *U* testing with Bonferroni correction for multiple comparisons.

The primary and secondary study endpoints were CIN3+ (including CIN3, AIS, SCC, and ADC) and CIN2+, respectively. The QIAsure Methylation Test was classified as positive when ΔCt values surpassed the threshold for methylation positivity defined by the manufacturer and validated in previous studies [12, 15, 18]. This threshold is hereafter referred to as the QIAsure Methylation Test threshold. The clinical performance of the *FAM19A4/miR124‐2* methylation using the QIAsure Methylation Test threshold was evaluated in terms of sensitivity and specificity. Specificity was calculated among all women without evidence of CIN2+, including women with CIN1, no CIN and “no histology.” Additionally, positive predictive value (PPV), negative predictive value (NPV), and colposcopy referral rates were calculated. For reference, the performance of baseline cytology (threshold ASC‐US) is provided although its sensitivity estimate is likely to be subject to verification bias. All estimates were calculated with corresponding 95% confidence intervals (CI) using the Wald method.

Alternative thresholds for *FAM19A4/miR124‐2* methylation analysis were explored in secondary analysis to adjust triage performance for use in primary HPV‐based screening. For this purpose, a multivariate logistic regression analysis was performed on the log_2_‐transformed ΔΔCt ratios of *FAM19A4* and *miR124‐2*. For the logistic regression analysis, 293 CIN3, 29 AIS, and 11 cervical cancers were used as cases, and 3026 samples of women without evidence of CIN2+ were used as controls. CIN2 cases were excluded from the training set due to their heterogenous nature. Diagnostic accuracy for CIN3+ was evaluated using receiver operating characteristic (ROC) curves and the area under the curve (AUC) value. Thresholds were set to predefined specificities of 70% and 80%. Clinical performance was assessed based on sensitivity, specificity, predictive values, and colposcopy referral rates, as outlined above on the full cohort, including CIN2 samples.

All statistical analyses were performed using R studio software (version 4.2.1, Vienna, Austria), including packages pROC and ggplot2. Figures were created in R Studio, GraphPad Prism (version 10.2.0, Boston, United States), and BioRender.

## 3 Results

### 3.1 Study Population

**FIGURE 1:**
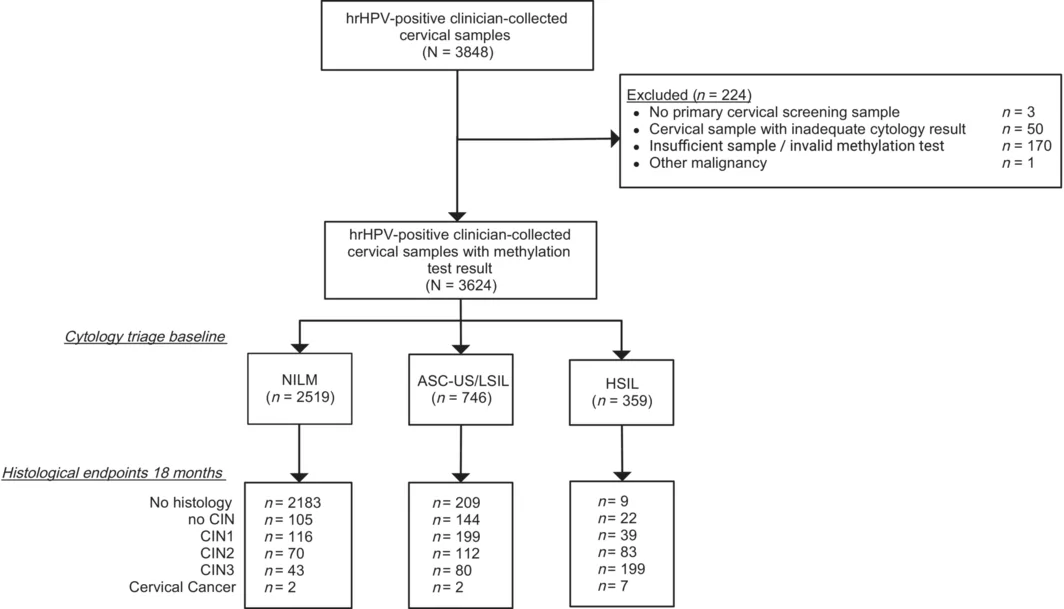
Study flowchart showing baseline cytology and histological endpoints after 18 months. ASC‐US/LSIL, atypical squamous cells of undetermined significance/low‐grade squamous intraepithelial lesion; CIN, cervical intraepithelial neoplasia grade; hrHPV, high‐risk human papillomavirus; HSIL, high‐grade squamous intraepithelial lesion; *N*, number; *n*, number of; NILM, negative for intraepithelial lesion or malignancy. Adenocarcinoma in situ is categorized as CIN3.

A consecutive series of 3848 hrHPV‐positive clinician‐collected cervical screening samples was collected between December 2020 and April 2021 (Figure 1). Of these, 224 (5.8%) samples were excluded from further analysis for the following reasons: not representing a primary cervical screening sample (*n* = 3), inadequate cytology result (*n* = 50), insufficient leftover sample for valid methylation analysis (*n* = 170) or endometrial cancer (*n* = 1). This resulted in a final series of 3624 hrHPV‐positive clinician‐collected cervical samples. The median age of the screening participants was 40 years (interquartile range 30–50). Baseline cytology results were normal (NILM) in 2519 women (69.5%), ASC‐US/LSIL in 746 women (20.6%) and HSIL in 359 women (9.9%). A total of 322 CIN3 cases, including 29 AIS, and 11 cervical cancer cases, including 4 ADCs and 7 SCCs, were detected after cytological triage during an 18‐month follow‐up period. The “No histology” group included 2171 women with 2× NILM and 230 women without a follow‐up sample.

### 3.2 DNA Methylation Levels of *FAM19A4* and *miR124‐2*

**FIGURE 2:**
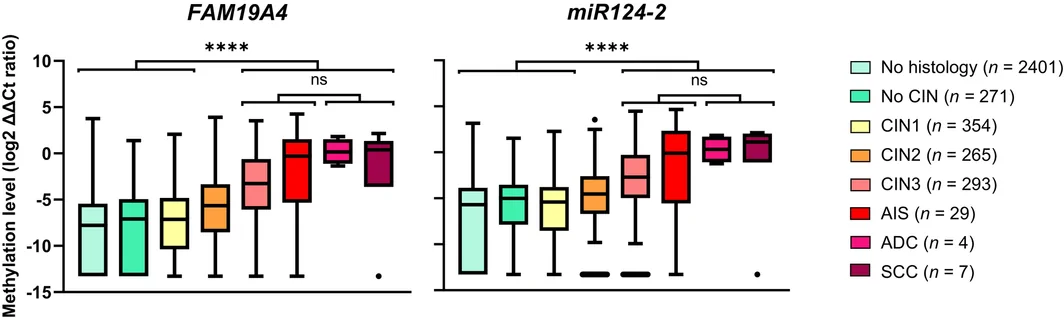
DNA methylation levels of *FAM19A4* and *miR124‐2* across histological endpoints. Methylation levels are shown as log2‐transformed ΔΔCt ratios. Boxplots show the median and interquartile range; whiskers extend to 1.5× the interquartile range. Outliers are shown as black dots. Differences between categories were tested by the Kruskal–Wallis test, followed by pairwise post hoc Mann–Whitney *U* testing with Bonferroni correction for multiple comparisons; ns, not significant (*p* > 0.05); ****p < 0.0001. ADC, adenocarcinoma; AIS, adenocarcinoma in situ; CIN, cervical intraepithelial neoplasia; Ct, cycle threshold; *n*, number of; SCC, squamous cell carcinoma.

Methylation levels of both *FAM19A4* and *miR124‐2* in hrHPV‐positive cervical screening samples showed a significant increase with underlying histological severity (*p* < 0.0001, Kruskal–Wallis Test; Figure 2). Methylation levels in CIN3+ (i.e., comprising CIN3, AIS, and cancer) were significantly higher compared to the levels in women with CIN1 or less (both markers *p* < 0.0001, Mann–Whitney *U* with Bonferroni correction). No significant differences were observed in methylation levels between CIN3/AIS and cancer (ADC/SCC) for either *FAM19A4* (*p* > 0.99) or *miR124‐2* (*p* = 0.88), nor differed the methylation levels of *FAM19A4* and *miR124‐2* between high‐grade glandular (AIS and ADC) and squamous (CIN3 and SCC) lesions (both markers *p* > 0.99). CIN2 cases exhibited intermediate methylation levels of both markers, being significantly different compared to both women with CIN1 or less, and women with CIN3+ lesions (*p* < 0.0001).

### 3.3 Performance QIAsure Methylation Test Threshold

The triage performance of *FAM19A4/miR124‐2* methylation using the QIAsure Methylation Test threshold for endpoints CIN3+ and CIN2+ can be found in Table 1. In this population‐based primary HPV‐based screening cohort, 56.2% (181/322) of cervical samples from women with CIN3/AIS (i.e., 55.3% (162/293) CIN3 and 65.5% (19/29) AIS) and 90.9% (10/11) of women with cervical cancer (i.e., 85.7% (6/7) SCC and 100% (4/4) ADC tested methylation positive) (Table 1). The sensitivity and specificity for detecting CIN3+ were 57.4% (95% CI: 52.0–62.7) and 88.3% (95% CI: 87.2–89.5), respectively. For reference, the performance estimates of baseline cytology are shown in Table 1.

**TABLE 1:** Clinical performance of *FAM19A4/miR124‐2* methylation using the QIAsure Methylation Test threshold and cytology (threshold ASC‐US) in high‐risk HPV‐positive women.

	*FAM19A4/miR124‐2* methylation		Cytology	
	QIAsure Methylation Test threshold		Threshold ASC‐US^a^	
	%	(*n*/*N*)	%	(*n*/*N*)
Positivity rate				
SCC	85.7%	(6/7)	85.7%	(6/7)
ADC	100.0%	(4/4)	75.0%	(3/4)
CIN3	55.3%	(162/293)	86.3%	(253/293)
AIS	65.5%	(19/29)	89.7%	(26/29)
CIN2	29.4%	(78/265)	73.6%	(195/265)
CIN1 or less^b^	11.7%	(353/3026)	20.6%	(622/3026)

	%	95% CI	%	95% CI
Sensitivity				
CIN3+	57.4%	(52.0–62.7)	86.5%	(82.8–90.2)
CIN2+	45.0%	(41.0–49.0)	80.8%	(77.6–83.9)
Specificity				
CIN1 or less^b^	88.3%	(87.2–89.5)	79.4%	(78.0–80.9)
PPV				
CIN3+	30.7%	(24.2–37.2)	26.1%	(21.0–31.1)
CIN2+	43.2%	(37.3–49.2)	43.7%	(39.3–48.1)
NPV				
CIN3+	95.3%	(94.5–96.0)	98.2%	(97.7–98.7)
CIN2+	89.0%	(87.9–90.2)	95.4%	(94.6–96.3)
Colposcopy referral				
Total	17.2%	(15.9–18.4)	30.5%	(29.0–32.0)

*Note:* Adenocarcinoma in situ is included in CIN3+.

Abbreviations: ADC, adenocarcinoma; AIS, adenocarcinoma in situ; ASC‐US, atypical squamous cells of undetermined significance; CI, confidence interval; CIN, cervical intraepithelial neoplasia; HPV, human papillomavirus; *N*, number; *n*, number of; NPV, negative predictive value; PPV, positive predictive value; SCC, squamous cell carcinoma.

^a^ The performance of baseline cytology is vulnerable to verification bias, given that high‐risk HPV‐positive women were managed by cytology in the Dutch cervical screening program.

^b^ CIN1 or less comprises all women without evidence of CIN2+, including women with CIN1, no CIN and “no histology.”

### 3.4 Diagnostic Prediction Model *FAM19A4/miR124‐2* Methylation

**FIGURE 3:**
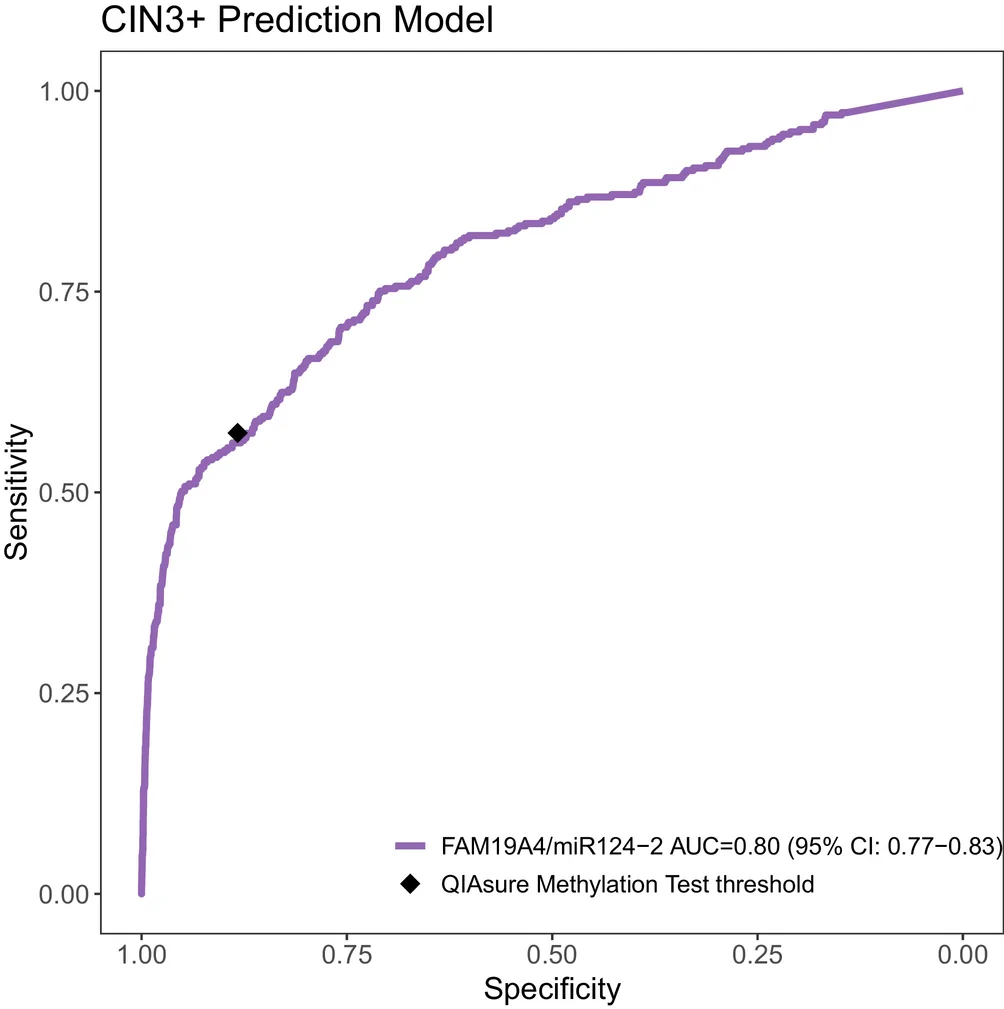
ROC curve and AUC value of the *FAM19A4/miR124‐2* prediction model for CIN3+ detection in high‐risk HPV‐positive clinician‐collected cervical samples. Point estimate for the performance of *FAM19A4/miR124‐2* methylation using the QIAsure Methylation Test threshold is indicated with ♦. AUC, area under the curve; CI, confidence interval; CIN, cervical intraepithelial neoplasia; HPV, human papillomavirus; ROC, receiver operating characteristics.

To optimize the thresholds of *FAM19A4/miR124‐2* methylation for application in population‐based primary HPV‐based screening, a logistic regression analysis was performed. The AUC value for detecting CIN3+ of the prediction model based on methylation of *FAM19A4* and *miR124‐2* was 0.80 (95% CI: 0.77–0.83) (Figure 3).

Using predefined thresholds corresponding to 70% and 80% specificity (Table 2), the *FAM19A4*/*miR124‐2* methylation model detected 74.8% and 65.2% (241/322 and 210/322, respectively) of CIN3/AIS cases overall, identifying 74.4% and 63.8% (218/293 and 187/293, respectively) of CIN3 cases, as well as 79.3% (23/29) of AIS cases. At both thresholds, 90.9% (10/11) of cervical cancer cases (i.e., 85.7% (6/7) CIN3 and 100% (4/4) AIS) were methylation positive (Table 2). The PPV for CIN3+ was 19.4% (95% CI: 14.5–24.3) and the NPV 96.5% (95% CI: 95.7–97.2). Details on CIN2+ performance can be found in Table 2. Stratification by age demonstrated comparable sensitivity for detecting CIN3+ in women aged < 40 years (*n* = 1794) and women ≥ 40 years (*n* = 1830) when using methylation‐based triage, while specificity differed (Table 3). In contrast, for cytology the sensitivity for detecting CIN3+ differed between women aged < 40 and ≥ 40 years while specificity was comparable.

**TABLE 2:** Clinical performance of *FAM19A4/miR124‐2* methylation using predefined 70% and 80% specificity thresholds in high‐risk HPV‐positive clinician‐collected cervical samples.

	*FAM19A4/miR124‐2* methylation			
	Predef. spec. 70%		Predef. spec. 80%	
	%	(*n*/*N*)	%	(*n*/*N*)
Positivity rate				
SCC	85.7%	(6/7)	85.7%	(6/7)
ADC	100.0%	(4/4)	100.0%	(4/4)
CIN3	74.4%	(218/293)	63.8%	(187/293)
AIS	79.3%	(23/29)	79.3%	(23/29)
CIN2	50.6%	(134/265)	39.2%	(104/265)
CIN1 or less^a^	30.0%	(908/3026)	20.0%	(605/3026)

	%	95% CI	%	95% CI
Sensitivity				
CIN3+	75.4%	(70.7–80.0)	66.1%	(61.0–71.2)
CIN2+	64.4%	(60.5–68.2)	54.2%	(50.2–58.2)
Specificity				
CIN1 or less^a^	70.0%	(68.4–71.6)	80.0%	(78.6–81.4)
PPV				
CIN3+	19.4%	(14.5–24.3)	23.7%	(18.1–29.3)
CIN2+	29.8%	(25.2–34.3)	34.9%	(29.7–40.1)
NPV				
CIN3+	96.5%	(95.7–97.2)	95.8%	(95.0–96.6)
CIN2+	90.9%	(89.6–92.1)	89.8%	(88.6–91.0)
Colposcopy referral				
Total	35.7%	(34.1–37.2)	25.6%	(24.2–27.1)

*Note:* Adenocarcinoma in situ is included in CIN3+.

Abbreviations: ADC, adenocarcinoma; AIS, adenocarcinoma in situ; CI, confidence interval; CIN, cervical intraepithelial neoplasia; HPV, human papillomavirus; *N*, number; *n*, number of; NPV, negative predictive value; PPV, positive predictive value; Predef. Spec., predefined specificity; SCC, squamous cell carcinoma.

^a^ CIN1 or less comprises all women without evidence of CIN2+, including women with CIN1, no CIN and “no histology.”

**TABLE 3:** Age‐stratified performance of *FAM19A4/miR124‐2* methylation and cytology in high‐risk HPV‐positive clinician‐collected cervical samples.

Triage method	< 40 years				≥ 40 years			
	Sensitivity		Specificity		Sensitivity		Specificity	
	%	95% CI	%	95% CI	%	95% CI	%	95% CI
*FAM19A4/miR124‐2* methylation								
QIAsure Methylation Test threshold	58.7%	(52.0–65.3)	91.7%	(90.3–93.1)	55.2%	(46.5–63.9)	85.3%	(83.5–87.0)
Predefined 70% specificity threshold	74.5%	(68.6–80.4)	77.4%	(75.2–79.6)	76.8%	(69.4–84.2)	63.3%	(60.9–65.7)
Predefined 80% specificity threshold	68.3%	(61.9–74.6)	85.8%	(84.0–87.6)	62.4%	(53.9–70.9)	74.7%	(72.6–76.9)
Cytology^a^								
ASC‐US threshold	89.9%	(85.8–94.0)	78.0%	(75.9–80.2)	80.8%	(73.9–87.7)	80.7%	(78.8–82.7)

*Note:* Performance was assessed by sensitivity for CIN3, AIS, and cancer detection, and specificity for identifying women with CIN1 or less.

Abbreviations: AIS, adenocarcinoma in situ; ASC‐US, atypical squamous cells of undetermined significance; CI, confidence interval; CIN, cervical intraepithelial neoplasia; HPV, human papillomavirus.

^a^ The performance of baseline cytology is vulnerable to verification bias, given that high‐risk HPV‐positive women were managed by cytology in the Dutch cervical screening program.

## 4 Discussion

In this prospective observational study, we assessed the clinical performance of *FAM19A4/miR124‐2* methylation as a triage test for hrHPV‐positive clinician‐collected cervical samples from the Dutch national primary HPV‐based cervical screening program. To the best of our knowledge, this is the first prospective evaluation of the diagnostic accuracy of the *FAM19A4/miR124‐2* methylation in a large population‐based primary HPV‐based screening cohort.

Consistent with previous studies, the DNA methylation levels in cervical screening samples increased with underlying disease severity, with highest levels in samples from women with CIN3 and cervical cancer [14, 28, 29]. Of interest, both glandular and squamous high‐grade lesions and cervical cancers showed high methylation levels for both *FAM19A4* and *miR124‐2*.

*FAM19A4/miR124‐2* methylation testing using the QIAsure Methylation Test threshold demonstrated high specificity with moderate sensitivity to detect CIN3. *FAM19A4/miR124‐*methylation levels are generally high in advanced transforming CIN lesions associated with long‐standing hrHPV infections (≥ 5 years) [14, 30, 31], and a higher risk of progression, whereas *FAM19A4/miR124‐2* methylation negativity has been linked to CIN2/3 lesions more likely to regress spontaneously [20]. This is the first‐time evaluation of *FAM19A4/miR124‐2* methylation in a primary HPV‐based screening cohort. Former studies included hrHPV‐positive screening samples collected at the time that routine screening was cytology‐based [15, 28]. The shift from cytology‐based screening towards primary HPV testing may have led to increased detection of smaller and less advanced CIN2/3 lesions [32, 33], which could have contributed to a reduced overall CIN3 detection rate when using the validated threshold of the QIAsure Methylation Test.

By calibration of *FAM19A4/miR124‐2* methylation analysis for application in primary HPV‐based screening, a triage performance with CIN3+ sensitivities of 75.4% (95% CI: 70.7–80.0) and 66.1% (95% CI: 61.0–71.2) at predefined specificities of 70% and 80%, respectively, was achieved. At these thresholds, *FAM19A4/miR124‐2* methylation was more sensitive for CIN3, AIS, and CIN2 than using the validated threshold of the QIAsure Methylation Test, while retaining reasonable specificity. The number of detected cancers, both SCC and ADC, remained similarly high between all three thresholds. In addition, comparable sensitivity for CIN3+ in women aged < 40 years compared to those aged ≥ 40 years was found for the different thresholds, while specificity was higher in the younger age group. The lower specificity observed in women aged ≥ 40 years may be attributed to higher background methylation levels associated with aging [34]. Overall, our findings indicate that *FAM19A4/miR124‐2* methylation appears to effectively stratify hrHPV‐positive women into higher‐ and lower‐risk groups, and can potentially redirect resources to the women with more advanced CIN lesions, who most benefit from direct referral and treatment. The extent of this benefit depends on the threshold chosen in a primary HPV screening setting, and hrHPV‐positive, methylation‐negative women may be managed through repeat testing. By using a longitudinal screening approach, the screening program will account for the natural history of cervical disease. Lesions not detected at initial triage could be early‐onset and may regress spontaneously, or will be detected at repeat testing in case of persistent or progressive lesions, thereby maintaining overall program safety. If direct triage is preferred, for example, in case of low adherence to follow‐up, a combination of methylation‐based triage with HPV genotyping may be applied, offering higher sensitivity for CIN3+ [11, 21, 35], although at the cost of specificity. Many HPV assays have integrated genotyping, at least partial (i.e., HPV16 and HPV18) or extended, making this an elegant combined baseline triage strategy. Although the Cobas HPV Test used in the Dutch screening program intrinsically performs HPV16/18 genotyping, HPV genotyping was only implemented for the triage of hrHPV‐positive women with ASC‐US or LSIL cytology in the Dutch screening program since 2022. Before 2022, HPV genotyping results were not recorded, and therefore these data were not available for the present study, preventing evaluation of the potential of HPV16/18 genotyping as an alternative or additive triage strategy.

The large cohort and real‐world setting provide a robust foundation for performance evaluation and calibration to an HPV‐based screening setting. A limitation of this study is verification bias of cytology, the triage standard at the time of this study. Cytology was applied selectively for disease verification as per screening protocol. Verification bias may have resulted in an overestimation of cytology sensitivity and an underestimation of the clinical performance of the methylation markers. Our results warrant further investigation of methylation‐based triage in prospective studies using methylation test results to guide referral within primary HPV‐based screening. Furthermore, the performance of cytology is prone to observer bias, since HPV status was known at the time of scoring [32, 33, 36]. Although comparison with cytology must be interpreted with caution, it is noteworthy that *FAM19A4*/*miR124‐2* methylation analysis, across all three thresholds used, detected 10 of 11 cervical cancer cases, including one woman who had NILM cytology at baseline. In addition, the sensitivity of cytology for detecting CIN3+ declined with age, whereas the sensitivity of methylation‐based triage remained comparable between younger and older age categories. Finally, given that this evaluation reflects the Dutch cervical screening program, extrapolation of the results to other settings should be done with caution. Quality of cytology varies widely among countries and the Netherlands outstands with a well‐established cytology infrastructure and extensive quality assurance [37]. The advantages afforded by *FAM19A4*/*miR124‐2* methylation‐based triage may therefore become more evident in settings with lower performance of cytology triage. As an objective molecular assay, methylation‐based triage minimizes interobserver variability, enhances reproducibility [12], and facilitates implementation in settings where healthcare resources are stressed or limited.

In this study we applied a direct bisulfite conversion treatment [26], providing a practical workflow with performance comparable to the protocol of DNA isolation followed by bisulfite treatment that was used in previous studies evaluating *FAM19A4*/*miR124‐2* methylation [15, 28]. Performing direct bisulfite treatment is advantageous, as it can be automated and is thereby applicable for high‐throughput implementation in routine cervical screening. Another advantage of methylation analysis is that it can be reliably performed on self‐collected samples [38, 39, 40], enabling a fully molecular screening strategy using a single self‐collected sample. Nowadays, HPV self‐sampling is used by more than half of the women participating in the Dutch screening program [41]. Methylation‐based triage will simplify the triage algorithm by eliminating the need for women who test hrHPV‐positive on a self‐sample to visit the general practitioner for a cervical sample for cytology triage. This may substantially reduce loss to follow‐up after a hrHPV‐positive self‐sampling test. It may also increase participation, since screening may become more attractive when it can be completed using a single self‐collected sample.

In conclusion, this study evaluated the performance of *FAM19A4*/*miR124‐2* methylation as a triage tool for hrHPV‐positive women in a real‐world, primary HPV‐based cervical screening setting. Our findings support the use of methylation‐based triage testing in primary HPV‐based screening programs, provided there is an adequate follow‐up policy in place for hrHPV‐positive, methylation‐negative women. Future studies are warranted to validate our findings in independent primary HPV‐based screening cohorts.

## Author Contributions

**Lisanne Verhoef:** data curation, methodology, writing – original draft, investigation. **Mila S. Griffioen:** data curation, investigation, writing – original draft, formal analysis, methodology, visualization. **Maaike C. G. Bleeker:** project administration, conceptualization, methodology, writing – review and editing. **John W. J. Hinrichs:** resources, writing – review and editing. **Albert G. Siebers:** resources, data curation, writing – review and editing. **Albertus T. Hesselink:** resources, writing – review and editing. **Chris J. L. M. Meijer:** conceptualization, methodology, writing – review and editing. **Renske D. M. Steenbergen:** conceptualization, methodology, funding acquisition, writing – review and editing. **Johannes Berkhof:** project administration, conceptualization, methodology, funding acquisition, writing – review and editing, formal analysis. **Daniëlle A. M. Heideman:** project administration, supervision, conceptualization, methodology, funding acquisition, writing – review and editing.

## Funding

This work was supported by the Horizon 2020 research and innovation program of the European Commission (RISCC project, grant agreement No. 847845). Mila S. Griffioen was supported by NWO‐KWF (grant KICH2.V4P.22.017). The QIAsure Methylation Test reagents and bisulfite conversion kits were kindly provided by QIAGEN (Hilden, Germany). The funding sources did not have any influence on the design of the study nor on analysis of the results.

## Ethics Statement

FSB (Facilitaire Samenwerking Bevolkingsonderzoeken, currently known as BVO‐NL (Utrecht, the Netherlands), the National Institute for Public Health and Environment (Bilthoven, the Netherlands), and the Medical Ethical Committee of Amsterdam UMC, location VU University Medical Center (Amsterdam, the Netherlands)) granted approval before start of the study (TcB2020‐245 and U2020‐051). In accordance with the Dutch screening program's protocol, all participants were informed that residual material might be used for anonymous research, with the option to opt out. Only leftover material from women who did not opt out was selected, stored, and analyzed after pseudonymization.

## Conflicts of Interest

(1) D.A.M.H., C.J.L.M.M., and R.D.M.S. are minority shareholders of Self‐screen B.V., a spin‐off company of VUmc; (2) Self‐screen B.V. develops, manufactures and licenses high‐risk HPV and methylation marker assays for cervical screening and holds patents on these tests; (3) R.D.M.S. declares consultancy fees from AstraZeneca paid to her institution; (4) D.A.M.H. reports grants from KWF (grant number 11337) and is member of the committee for assessment of molecular diagnostics (CieBOD) of the Dutch Society of Pathology (NVVP) with honorarium paid to her institution; (5) A.T.H. is an employee of Self‐screen B.V.; (6) C.J.L.M.M. is part time CEO of Self‐screen and serves occasionally on the scientific advisory board of QIAGEN; (7) L.V., M.S.G., M.C.G.B., J.W.J.H., A.G.B., and J.B. declare no conflicts of interest.

## Data Availability

The data that support the findings of our study are available from the corresponding author upon reasonable request.
